# Correction: Voluntary sports programs for individuals with mental health disorders: The trainer’s view

**DOI:** 10.1371/journal.pone.0315686

**Published:** 2024-12-09

**Authors:** Florence Epiney, Frank Wieber, Daniela Loosli, Hansjörg Znoj, Nikolai Kiselev

There are errors in the author affiliations. The correct affiliations are as follows:

Florence Epiney^1,2^, Frank Wieber^3,4^, Daniela Loosli^6^, Hansjörg Znoj^2^, Nikolai Kiselev^5,6^

**1** PluSport Bern Gruppen, Bern, Switzerland, **2** Department of Psychology, University of Bern, Bern, Switzerland, **3** School of Health Sciences, Institute of Public Health, Zurich University of Applied Sciences ZHAW, Winterthur, Switzerland, **4** Department of Psychology, University of Konstanz, Konstanz, Germany, **5** Swiss Research Institute for Public Health and Addiction (ISGF), University of Zurich, Zurich, Switzerland, **6** PluSport, Umbrella Organization of Swiss Disabled Sports, Volketswil, Switzerland.
