# Supplementary material for: Voluntary sports programs for individuals with mental health disorders: The trainer’s view
Source: PLoS One. 2023 Oct 26;18(10):e0290404. doi: 10.1371/journal.pone.0290404 (PMC10602351; doi:10.1371/journal.pone.0290404)
Supplement: Attachment — Submitted filename: Revisions_Artikel_PSY-Trainers.docx [file pone.0290404.s001.docx]

Rogis Baker, Ph.D

Academic Editor

PLOS ONE

Volketswil, 25. Juni 2023

**→ Revisions for the manuscript:**

**Voluntary sports programs for individuals with mental health disorders: the trainer's view**

Dear Dr. Baker

Thank you very much for the time and effort you, the anonymous first reviewer, and Mr. Dr. Mohammed Feroz Ali as the second reviewer, have spent reviewing our manuscript "*Voluntary sports programs for individuals with mental health disorders: the trainer's view*".

We appreciate the opportunity to revise and resubmit our manuscript to your journal, *PLOS ONE*.

Please find below the reviewers' comments (bold/italic) with our responses, including how and where the text was modified point by point. Additionally, a new version of the manuscript (with accepted revisions) and a "track changes" version are attached.

All authors have read and approved the revision of the manuscript.

Thank you very much for considering this manuscript.

Sincerely,

Nikolai Kiselev (on behalf of the authors)

**REVISIONS ON MANUSCRIPT**

**REVIEWER 1 COMMENTS**

***Interesting article.***

Answer:	Dear reviewer, thank you for this feedback as well as your valuable time reviewing our manuscript and providing your further recommendations.

1. ***In methodology - suggest to describe about the interviewer in more detail. E.g. how many interviewer, which year are they, are they homogenous group of students and are they part of the study team***

Answer:	Thank you for this advice. We extended the information section about the interviewer in the section "2.3. Interviewer". At the same time, we would like to note that we only had one interviewer for all the interviews.

2. ***Recruitment- Please explain or reword "corona time"***

Answer:	We agree that corona time is imprecise. We changed it to "the period impacted by the coronavirus outbreak and related restrictions" (section "2.4. Recruitment")

3. ***Do you apply for ethic clearance?***

Answer:	Given Switzerland's official regulation, our study does not need ethical clearance. But it was checked and approved by the ethics committee of the University of Zurich. We added this statement to the section "2.7. Ethics".

4. ***I don't think you need to mention regarding the gift etc in the text***

Answer:	Thank you for you opinion. We deleted the part regarding the gift (section "2.5. Process").

5. ***Please reword/ rephrase "Msc in psychology" not everyone familiar with that***

Answer:	Thank you for this advice. We changed it to "a student in the last year of the study program for the master's degree in psychology" (section "2.6. Analysis").

6. ***Table 1: spelling mistake***

Answer:	Thank you for paying attention to the details. We corrected the spelling mistakes in the Table 1.

**REVIEWER 2 COMMENTS**

***The article has good data […],***

Answer:	Dear Dr. Mohammed Feroz Ali, we appreciate your feedback and recommendation on our article. Thank you for your time you dedicated to review it.

a. ***[…] although the authors have said that they done need ethical approval for such publications i would recommend that ethical approval be taken.***

Answer:	Given Switzerland's official regulation, our study does not need ethical clearance. But it was checked and approved by the ethics committee of the University of Zurich. We added this statement to the section "2.7. Ethics".

b. ***The authors are advised to add some recommendations based on the finding.***

Ânswer:	Thank you for this advice. We added a new chapter with recommendations based on findings and discussion (chapter "6. Current recommendations").
